# A 3D reconstruction platform for complex plants using OB-NeRF

**DOI:** 10.3389/fpls.2025.1449626

**Published:** 2025-03-10

**Authors:** Sixiao Wu, Changhao Hu, Boyuan Tian, Yuan Huang, Shuo Yang, Shanjun Li, Shengyong Xu

**Affiliations:** ^1^ China College of Engineering/Key Laboratory of Agricultural Equipment for the Middle and Lower Reaches of the Yangtze River, Ministry of Agriculture, Huazhong Agricultural University, Wuhan, China; ^2^ College of Horticulture and Forestry Sciences, National Key Laboratory for Germplasm Innovation & Utilization of Horticultural Crops, Huazhong Agricultural University, Wuhan, China; ^3^ Xianning Academy of Agricultural Science, Xianning, China

**Keywords:** neural radiance fields, 3D reconstruction, plant phenotyping, digital twins, mesh

## Abstract

**Introduction:**

Applying 3D reconstruction techniques to individual plants has enhanced high-throughput phenotyping and provided accurate data support for developing "digital twins" in the agricultural domain. High costs, slow processing times, intricate workflows, and limited automation often constrain the application of existing 3D reconstruction platforms.

**Methods:**

We develop a 3D reconstruction platform for complex plants to overcome these issues. Initially, a video acquisition system is built based on "camera to plant" mode. Then, we extract the keyframes in the videos. After that, Zhang Zhengyou's calibration method and Structure from Motion(SfM)are utilized to estimate the camera parameters. Next, Camera poses estimated from SfM were automatically calibrated using camera imaging trajectories as prior knowledge. Finally, Object-Based NeRF we proposed is utilized for the fine-scale reconstruction of plants. The OB-NeRF algorithm introduced a new ray sampling strategy that improved the efficiency and quality of target plant reconstruction without segmenting the background of images. Furthermore, the precision of the reconstruction was enhanced by optimizing camera poses. An exposure adjustment phase was integrated to improve the algorithm's robustness in uneven lighting conditions. The training process was significantly accelerated through the use of shallow MLP and multi-resolution hash encoding. Lastly, the camera imaging trajectories contributed to the automatic localization of target plants within the scene, enabling the automated extraction of Mesh.

**Results and discussion:**

Our pipeline reconstructed high-quality neural radiance fields of the target plant from captured videos in just 250 seconds, enabling the synthesis of novel viewpoint images and the extraction of Mesh. OB-NeRF surpasses NeRF in PSNR evaluation and reduces the reconstruction time from over 10 hours to just 30 Seconds. Compared to Instant-NGP, NeRFacto, and NeuS, OB-NeRF achieves higher reconstruction quality in a shorter reconstruction time. Moreover, Our reconstructed 3D model demonstrated superior texture and geometric fidelity compared to those generated by COLMAP and Kinect-based reconstruction methods. The $R^2$ was 0.9933,0.9881 and 0.9883 for plant height, leaf length, and leaf width, respectively. The MAE was 2.0947, 0.1898, and 0.1199 cm. The 3D reconstruction platform introduced in this study provides a robust foundation for high-throughput phenotyping and the creation of agricultural “digital twins”.

## 1 Introduction

3D reconstruction of plants facilitates the high-throughput phenotypic and the realization of “digital twins” in agriculture (Kim and Heo, 2024; Peladarinos et al., 2023; Pylianidis et al., 2021). High-throughput phenotypic analysis is an essential component of plant science and plays a significant role in agricultural production and genotype-phenotype studies. In agricultural production, the revolutionary integration of emerging sensors with artificial intelligence (AI) has transformed precision agriculture (PA), significantly enhancing the efficiency, effectiveness, and productivity of agricultural industry breeding and primary production (Sishodia et al., 2020). Consequently, the comprehensive analysis of plant phenotypes for monitoring plant growth has become increasingly crucial (Fu et al., 2020). In the field of genotype-phenotype studies, recent years have seen tremendous advances in plant genome sequencing, propelling the research of crop improvement that combines genotyping with phenotyping (Sun et al., 2022). However, progress in phenotypic analysis has been slow, hindering development. Traditional phenotypic methods largely rely on manual measurements, which are time-consuming, labor-intensive, and cannot guarantee accuracy. Furthermore, manual measurements cannot track the entire plant lifecycle comprehensively (Xiao et al., 2022). High-throughput phenotypic analysis based on 3D reconstruction overcomes the aforementioned drawbacks of traditional methods, emerging as a potent tool for evaluating plant phenotypes.

3D reconstruction technologies can be categorized into active and passive vision systems (Lu et al., 2020). Active vision is a reconstruction method that utilizes its light source to measure distances by projecting it onto the object. LiDAR and depth cameras are the mainstream active vision imaging devices. LiDAR is a commonly employed technique for acquiring 3D plant data (Luo et al., 2021; Tsoulias et al., 2023; Walter et al., 2019), renowned for its high precision and resolution; however, it is costly and time-consuming. In recent years, with Microsoft’s introduction of the cost-effective Kinect series of depth cameras, methods based on depth cameras for 3D reconstruction have been proposed (Song et al., 2023; Teng et al., 2021a; Yang et al., 2019). Nevertheless, the data resolution obtained from depth cameras is low, and due to the unique structure of plants, the 3D representation capability of depth cameras is significantly degraded by distortion and noise, this significantly hampers the accuracy of reconstructing individual plants, especially at the finer organ scale.

Passive vision systems employ cameras to capture images of objects, extracting 3D data through image analysis. Conventional passive vision techniques include Structure from Motion (SfM) paired with MultiView Stereo (MVS), as well as voxel carving. Voxel carving utilizes segmentation masks to reconstruct objects from various perspectives, and it has been widely adopted for 3D reconstruction and the phenotypic analysis of plants (Das Choudhury et al., 2020; Golbach et al., 2015; Scharr et al., 2017). However, voxel carving is generally confined to conventional plant science laboratory environments where multiple camera positions are static and can be precisely calibrated (Feng et al., 2023). Moreover, when applied to larger plants, such as maize, voxel carving can only proceed within a limited number of views. it struggles to robustly reconstruct extensive 3D structures from numerous angles (e.g., 15 or more), which compromises the quality of the reconstruction (Tross et al., 2021).

SfM-MVS is extensively utilized in the analysis of morphological and structural plant phenotypes and is acknowledged as an optimal approach for creating a high-throughput, cost-effective platform for individual plant phenotyping (Wu et al., 2022). Nevertheless, it exhibits certain drawbacks: (1) To improve the quality and efficiency of the reconstruction process, it is imperative to acquire plant masks and provide uniform illumination conditions (Wu et al., 2022). (Gao et al., 2021) (2) Since the camera’s intrinsic parameters and poses are derived through SfM, an additional calibration tool is essential to convert the algorithm-generated models into metric reconstructions (Li et al., 2022). (3) The time required for reconstruction is considerable.

In recent years, the advent of NeRF (Neural Radiance Fields) (Mildenhall et al., 2020), an innovative passive vision technology based on deep learning, has garnered considerable interest for its capacity to render high-fidelity reconstructions of intricate plant and agricultural environments (Hu et al., 2024). Traditional 3D reconstruction methodologies yield “explicit” 3D models, including point clouds, meshes, and voxel arrays (Schönberger and Frahm, 2016; Seitz et al., 2006). These “explicit” forms are inherently discrete, which can result in a loss of geometric and textural fidelity. In contrast, NeRF falls under the category of implicit neural representation methods, boasting the advantage of continuous implicit functions, which not only allow for the synthesis of new viewpoint images of plants but also enable the extraction of textured mesh models and colored point clouds. So, NeRF holds promise as a key bridge between two-dimensional imaging and 3D reconstruction—two methods of acquiring plant phenotypes. It thereby facilitates the establishment of low-cost, high-throughput, and non-destructive plant phenotyping analysis systems (Hu et al., 2024). Moreover, by leveraging this neural implicit representation, NeRF is capable of mapping real crops into a computational virtual space, facilitating a VR-style immersive interaction for users. This innovative method offers a new avenue for creating “digital twins” within the agricultural industry (Zhang et al., 2024). Despite its potential, NeRF demonstrates several limitations. 1. Reconstructing an individual plant with NeRF can take upwards of 10 hours. 2. It cannot automatically segregate a single plant from a neural radiance field that encompasses a background. 3. There is still room for improvement in NeRF’s ability to reconstruct highly detailed plant geometry. 4. Its performance is highly susceptible to lighting conditions (Cui et al., 2024).

As an early attempt in the field of 3D reconstruction of individual plants using NeRF, a new 3D reconstruction platform based on Object-Based NeRF(OB-NeRF) is presented for complex plants such as citrus fruit tree seedlings, this study makes the following contributions:

1. We propose an improved version of the NeRF model, called Object-Based NeRF (OB-NeRF), which enables high-throughput, automated, and high-precision 3D reconstruction of individual plants under complex backgrounds and uneven lighting conditions.
2. We propose a camera poses global calibration strategy, which uses the predetermined camera imaging trajectory as prior information to automatically calibrate the camera pose so that the pose and size of the reconstructed plant model can be restored to the true value.
3. A plant reconstruction platform based on the multi-view images to reconstruct high-precision and proportional mesh models for complex plants is constructed, integrating a “camera to plant” video acquisition system, as well as algorithms for Keyframe extraction, estimation and calibration of camera parameters, and 3D reconstruction based on OB-Nerf.

## 2 Materials

**Figure 1:**
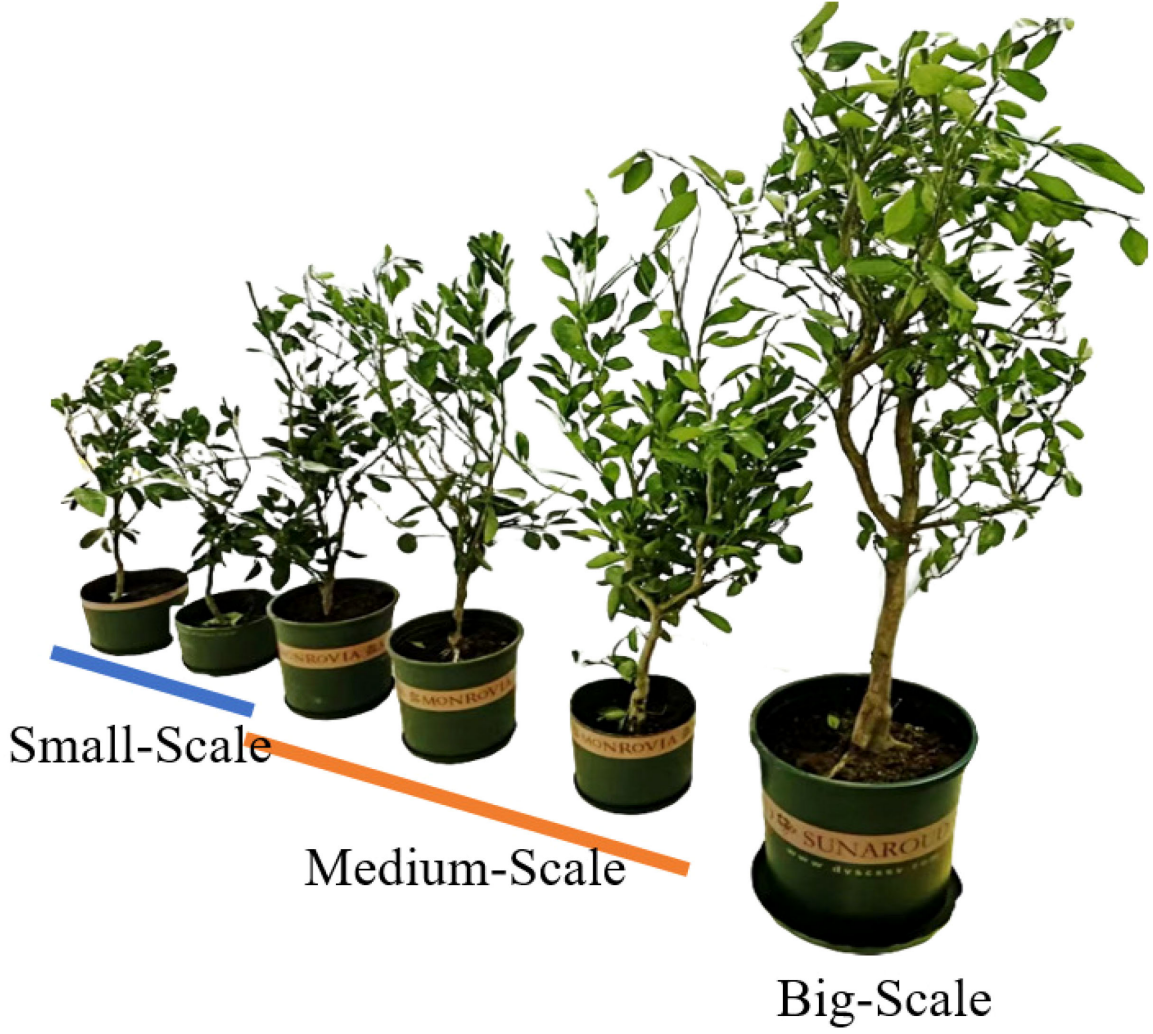
Experimental materials.

The saplings used in this study were obtained from the digital orchard demonstration base at Huazhong Agricultural University(114°366403’N, 22°756488’E). The variety selected was the mandarin orange (Citrus reticulata ‘Yura’), a species widely cultivated in China. The ages of the saplings ranged from 6 months to 2 years, with an average age of 18 months. The average height of the saplings was 120 cm. All saplings were grown under the same standard management practices, which included regular irrigation, fertilization, and pest control. As shown in **Figure 1**, we categorized saplings into three groups based on their height: the small size group with heights ranging from 60 to 90 cm, the medium size group from 90 to 130 cm, and The large-size group is defined as individuals with a height ranging from 130 cm to 170 cm. It was observed that as the saplings grew, the complexity of their canopy structure increased. The study included a total of 20 fruit tree saplings: 7 in the small-size group, 7 in the medium-size group, and 6 in the large-size group. The tree seedlings were transferred into flower pots before the experiment. the plant height, leaf width, and leaf width are initially recorded by manual measurement. Subsequently, a video acquisition system obtained multi-view videos of the plants, serving as the data source for subsequent experiments.

## 3 Methods

**Figure 2:**
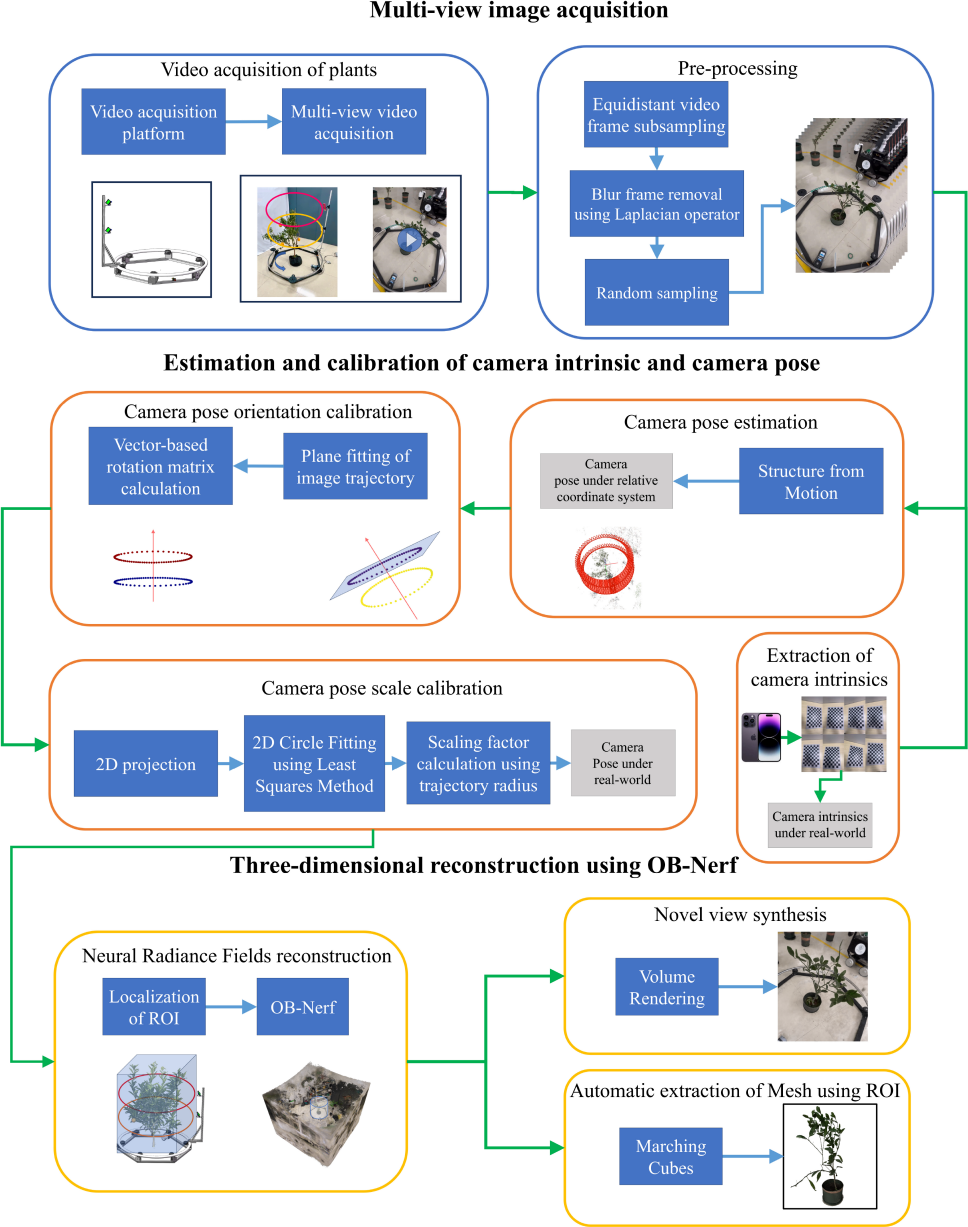
The pipeline of 3D reconstruction method.

The pipeline of the 3D reconstruction method proposed in this paper is shown in **Figure 2**. Firstly, an image acquisition system automatically captures Multi-view videos of plants. Then, transmit videos to the computer. Secondly, the Zhengyou Zhang calibration method (Zhang, 1999) is used to obtain the parameters of cameras. Moreover, the camera poses are estimated by SFM. Thirdly, the camera poses are calibrated by the camera poses global calibration strategy based on Hardware-Software Co-Design. Finally, we reconstruct the target plant’s neural radiance field using the proposed OB-Nerf algorithms and the Marching Cubes algorithm extracts the mesh model from the neural radiation field. The development and testing of the reconstruction pipeline were conducted on a computer equipped with an Intel Core i7-12700H processor, 16GB memory, and an NVIDIA GeForce RTX 3070Ti Laptop GPU. The computer operating system is Windows 11.

### 3.1 Multi-view images acquisition

#### 3.1.1 Video acquisition system

**Figure 3:**
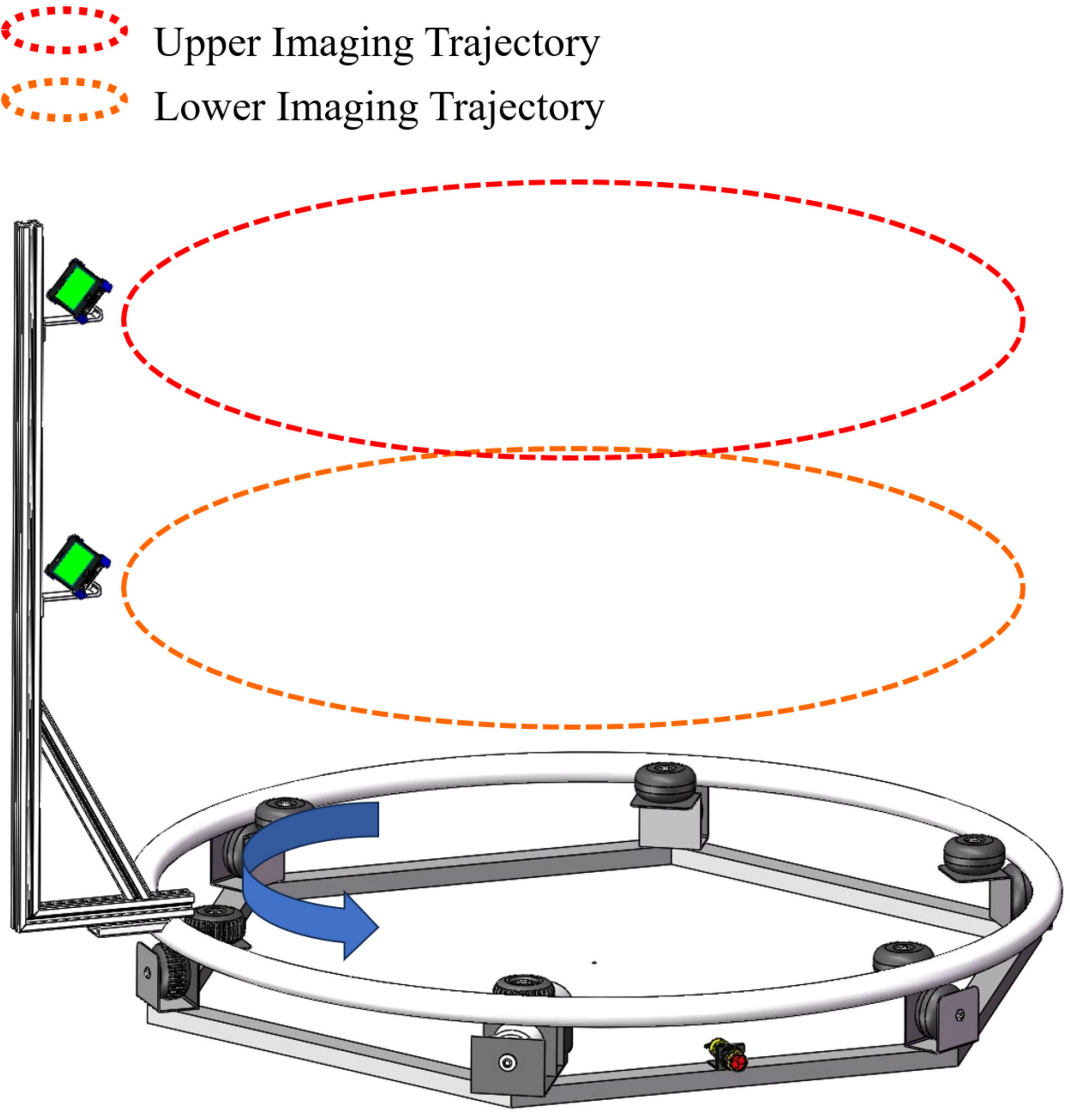
Hardware of video acquisition system.

An automated system for video acquisition is presented, as depicted in **Figure 3**. Our system comprises several components: a controller, limit switches, a hexagonal base, a direct current (DC) motor, a driven pulley, a metal ring, a camera bracket, a camera, and counterweights. Notably, the metal ring has a radius of 75 cm, and the base of the camera bracket is made from an aluminum profile with a slotted design. This design allows for the radius of the imaging trajectory to be adjustable, providing a range of up to 30 cm. The height of the two cameras is adjustable within a range of 0-1.5 meters. This flexibility enables us to adjust the radius and height of the camera’s imaging trajectory to accommodate plants of varying sizes. This paper proposes a linear procedure for video acquisition as follows:

##### 3.1.1.1 Device setup

The target plant is centrally positioned within the system. Adjusting the radius and camera height to accommodate the size of the target plant, we recorded the radius *r_real_*, the height of the lower camera from the ground *h*_1_, and the height *h*_2_ from the upper camera to the top of the bracket as prior knowledge for the reconstruction algorithm.

##### 3.1.1.2 Video capture

Once the system receives the start signal sent by the remote control, the DC motor drives the metal ring equipped with the camera bracket to rotate. The camera orbits the plant, capturing a 360-degree video. Once the video capture is complete, the DC motor automatically stops. Each capture is completed within 15 seconds. Our System is compatible with any standard RGB imaging device. For our experimental setup, we selected two iPhone 14 Pro smartphones, which offer a video resolution of 1080x1920 at a frame rate of 30fps.

#### 3.1.2 Keyframe extraction

In the Structure from Motion (SFM) process, we observe that the relationship between the number of images and the reconstruction time follows a quadratic function, with the reconstruction time increasing dramatically as the number of images increases. Insufficient image quantity and image blur both negatively impact the reconstruction quality when using OB-NeRF for reconstruction. Therefore, this research aims to find the optimal balance between reconstruction quality and time cost through a series of experiments, which are detailed in sections 4.2.1 and 4.2.2. The experimental results demonstrate that high-quality 3D reconstruction can be achieved in a relatively short time by excluding blurry frames and limiting the number of images to approximately 90, which is a significant improvement compared to using all available images. To achieve this, we develop an intelligent frame extraction algorithm that can automatically select 90 clear frames from the input video for subsequent reconstruction, effectively reducing the computational burden while maintaining the reconstruction quality. The algorithm workflow is as follows:

1. Perform equidistant downsampling on the binocular videos obtained by the video acquisition system to acquire 50 images from each perspective, totaling 100 images. Uniform downsampling can reduce the number of images and effectively decrease redundancy by avoiding the repeated selection of similar frames.
2. Eliminate blurry frames. To quantitatively assess the sharpness of the images, this study adopted a method based on the variance of the Laplacian operator. First, the RGB image is converted to grayscale, and then the Laplacian operator *L* is calculated using an 8-neighborhood convolution kernel.Finally, we calculate the variance of the resulting Laplacian image to obtain a variance value, which serves as a measure of sharpness. The greater the variance, the higher the image sharpness. The mathematical expression for variance is shown in Equation 1:

(1) $$\text{Var}(L)=\frac{1}{N}\sum_{i=1}^{N}{\left(L_{i}-\mu \right)}^{2}$$

We calculate the average variance of all images and eliminate those that are less than 20% of the average value. If the number of images removed exceeds 10, it is determined that the data quality is not up to standard, and image acquisition needs to be repeated.

3. To ensure a total of 90 images, we conduct a random sampling of the remaining images after eliminating blurry frames, extracting 45 images from each perspective, a total of 90 images.

To enhance efficiency, we employed multithreaded parallel computing techniques for equidistant downsampling and the removal of blurred frames. Consequently, the overall preprocessing duration was reduced to roughly 3 seconds.

### 3.2 Estimation and calibration of camera parameters

#### 3.2.1 Camera parameters estimation

The camera projection model defines the mapping from a 3D world to a 2D image plane. *A* is the camera intrinsic matrix, *R* and *t* are the rotation and translation, *k*_1_ *, k*_2_ *, k*_3_ are the distortion coefficients, that describe the change of coordinates from world to camera coordinate systems.

##### 3.2.1.1 Camera intrinsic parameters extraction

The precise camera projection model was established in this study using Zhang Zhengyou’s calibration method. The method involves the following steps: preparing a calibration board, capturing calibration images, extracting corners of the calibration board, establishing correspondences between corners, computing the camera’s intrinsic parameters, estimating distortion parameters, and Optimizing the parameters mentioned above with the L-M algorithm. We Prepared a chessboard with a grid size of 40 mm * 40 mm and dimensions of 12 × 9 corners and obtain the internal parameters $A=[\begin{array}{ccc} 872.2 & 0 & 540.6\\ 873.1 & 957.9\\ 0 & 0 & 1 \end{array}]$ and distortion coefficients *k*_1_ = 6.2 × 10^−3^, *k*_2_ = −3.95331432 × 10^−2^, *k*_3_ = 7.5 × 10^−5^, *p*_1_ = −1.63556310 × 10^−4^, *p*_2_ = 4.11907012 × 10^−2^.

##### 3.2.1.2 Camera pose estimation

SFM can estimate the camera poses, which are inputs to the OB-NeRF. The input of SFM includes a set of plant image sequences, and the camera intrinsic parameters. Initializing camera intrinsic parameters at the beginning of SFM provides a robust initial estimate, which reduces calculated errors, accelerates algorithmic convergence, and enhances the accuracy of pose estimation. SFM outputs the camera pose matrix corresponding to each image.

#### 3.2.2 Camera poses global calibration strategy based on Hardware-Software Co-Design

The camera poses obtained through the SFM are estimated within a relative coordinate system, hereafter referred to as the “virtual coordinate system”. Since the virtual coordinate system is not directly aligned with the real-world coordinate system, the reconstructed plant model’s dimensions and orientation may not match the real-world counterparts. Aligning the coordinate systems is crucial for accurate camera pose calibration, as it establishes the transformation relationship between the virtual coordinate system and the real-world coordinate system. The transformation relationship between coordinate systems encompasses both orientation and scale relationships. The orientation relationship is characterized by the rotation matrix *R_vtr_*. The scale relationship is denoted by the scaling factor *k*. The calibration formulae for the camera’s poses as delineated in Equations 2 and 3:


(2) $$R=R_{vtr}\cdot R_{\text{virtual}},t_{virtual}=R_{vtr}\cdot t_{virtual}$$

(3) $$t=k\cdot t_{\text{virtual}}$$

We propose an automatic camera pose global calibration strategy based on Hardware-Software Co-Design. It leverages the camera’s predetermined imaging trajectory as a reference. More precisely, the normal vector of the trajectory plane is utilized as a direction reference for the coordinate systems, Concurrently, the diameter of the trajectory is utilized as a scale reference for the coordinate systems. Moreover, the process of this method is as follows:

1. Orientation calibration of the camera pose using the Least squares method and Rodrigues’ rotation equation. The imaging trajectory is horizontal, suggesting that the normal vector of the trajectory plane is parallel to the z-axis of the real world. Initially, we use the camera positions *A* = {(*x*_1_ *, y*_1_ *, z*_1_),(*x*_2_ *, y*_2_ *, z*_2_)*,…*,(*x_n_, y_n_, z_n_*)} restored by SFM as a discrete representation of the camera imaging trajectory, fit the imaging trajectory to a plane using the Least squares method to acquire the normal vector (*a, b, c*). Thirdly, calculate the rotation matrix *R_vtr_* based on the normal vector (*a, b, c*) of the imaging trajectory plane and the direction vector (0, 0, 1) of the z-axis using Rodrigues’ rotation equation. Finally, apply the orientation calibration formulae to the camera poses.
2. Calculation of scale factor using imaging trajectory. The scale factor *k* is calculated based on the actual radius of the trajectory as prior information. First, we project the camera trajectory points after orientation calibration onto the XY plane. Secondly, fit the 2D trajectory points to a circle ${\left(x-x_{c}\right)}^{2}+\;{\left(y-y_{c}\right)}^{2}=r_{virtual}^{2}$ using the Least squares. Thirdly, calculate the scale factor *k* based on $r_{virtual}$ and the imaging trajectory’s real radius $r_{real}$ according to Equation 4. Finally, apply the scale calibration formula to the camera poses.

(4) $$k=\frac{r_{real}}{r_{virtual}}$$

Moreover, to accelerate the training process of OB-Nerf, we translate the coordinate system, thereby shifting the target plant to the center of the coordinate system. First, we separate the camera imaging positions of the upper and lower tracks by utilizing the height difference and calculate the average zcoordinate of the two-point sets, denoted as *z_up_* and *z_low_*. Second, we calculate the translation vector ${\vec{t}}_{s}$ according to Equation 5. Finally, apply the translation formula (Equation 6) to the camera poses.


(5) $$t_{s}={\left(x_{c},y_{c},z_{c}\right)}^{T}$$

Where: $z_{c}=\frac{z_{up}+z_{low}}{2}$ .


(6) $$t=t-t_{s}$$

### 3.3 3D reconstruction using object-based Nerf

#### 3.3.1 Localization of region of Interest

**Figure 4:**
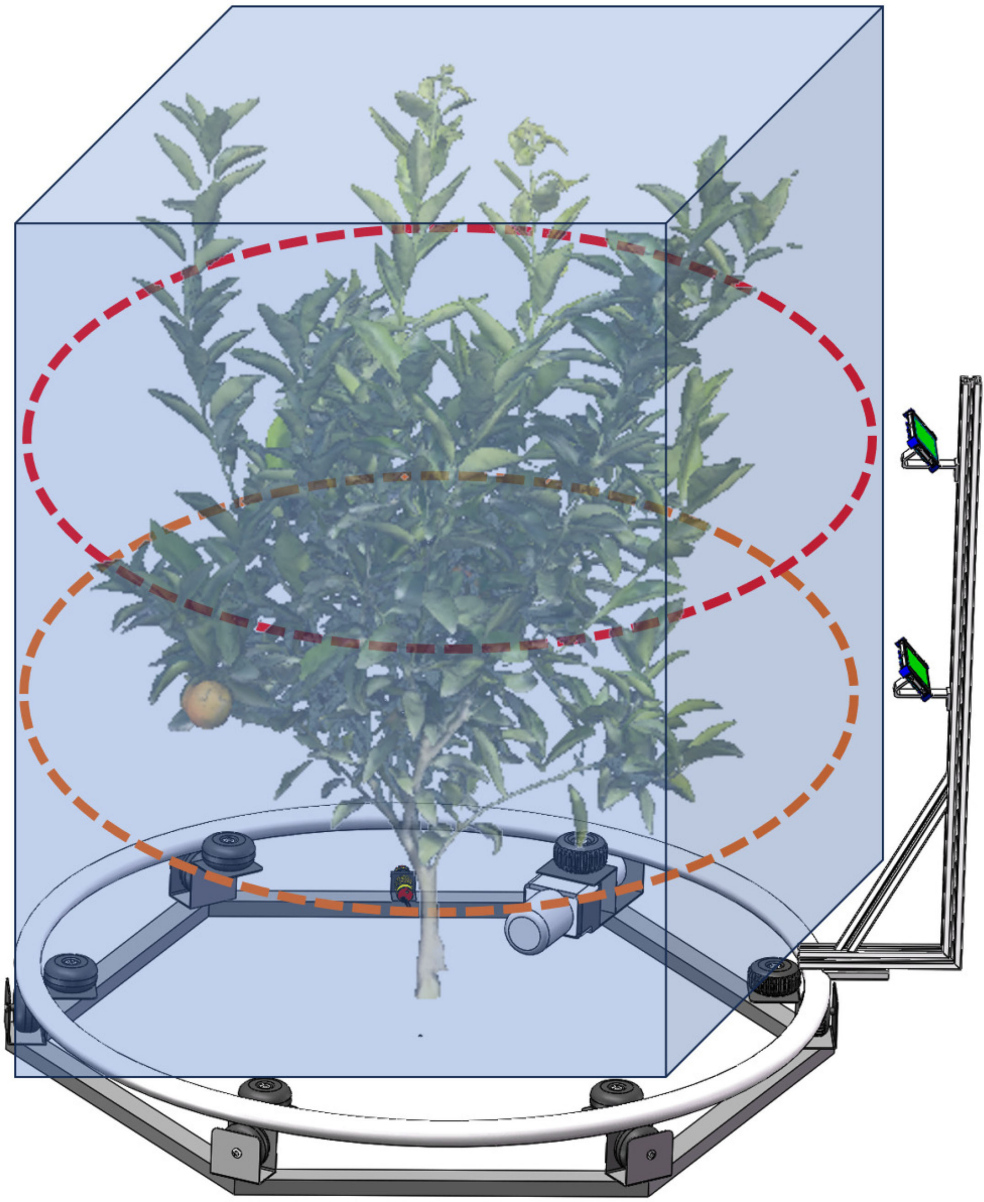
Location of the region of interest.


Traditional multi-view image-based plant 3D reconstruction methods typically utilize background removal to enhance reconstruction accuracy and computational efficiency. But in natural scenes, it poses a challenge, thus necessitating setting up a solid-colored studio, which is difficult and costly for “camera to plant” mode. To achieve high-quality reconstruction of the target plant without performing background segmentation, it is essential to determine the spatial region in which the plant is located within the scene. This region is called the Region of Interest (ROI), which acts as prior knowledge for the OB-Nerf algorithm (**Figure 4**). Due to its high computational efficiency in the ray sampling process, we use the axis-aligned bounding box (AABB) to represent the ROI. An AABB is a rectangular box defined by its minimum and maximum coordinates $\left(x_{\text{min}},y_{\text{min}},z_{\text{min}}\right)$ and $\left(x_{\text{max}},y_{\text{max}},z_{\text{max}}\right)$ , which are parallel to the coordinate axes. In section 3.3.2, we computed the heights of two imaging trajectories, *z_up_* and *z_low_*. The calculation formula for AABB as illustrated in Equation 7:

(7) $$\left\{ \begin{array}{l} \left(x_{\text{min}},y_{\text{min}},z_{\text{min}})=(-r_{real},-r_{real},z_{low}-h_{1}\right)\\ \left(x_{\text{max}},y_{\text{max}},z_{\text{max}})=(r_{real},r_{real},z_{up}+h_{2}\right) \end{array}\right.$$

#### 3.3.3 Object-based NeRF

Neural Radiance Fields implicitly represent 3D scenes through neural network approximations of 3D density fields and 5D color fields. The radiation field describes the volumetric density at every point in a scene, as well as the color of each point from every observational direction, expressible by the function: *F_θ_* : (*x,d*) → (*c, σ*). Initially, the light rays emitted from the camera are sampled to determine the positions of each sampling point. Subsequently, these positions are encoded and mapped into a high-dimensional space. Subsequently, these encoded inputs are fed into a multilayer perceptron, which is utilized to approximate the 3D density field, thereby obtaining the volume density corresponding to each point. Next, the volume density output is concatenated with the view direction vector, and these combined inputs are fed into another multilayer perceptron, designed to fit the 5D light field, thereby retrieving color for each point in the observed direction. Finally, the image is synthesized through volume rendering, and the network is optimized by minimizing the loss between the synthesized and actual view color values using backpropagation. This study proposes targeted enhancement strategies to address the issues encountered by the NeRF model in plant 3D reconstruction:

1. A critical requirement for achieving high-throughput reconstruction is completing the process of reconstructing individual plants within a short time frame. We have employed a shallow multilayer perceptron to approximate the density and color fields.
2. In phenotyping, reconstructing individual plants requires high fidelity not only at the macroscopic level but also at the organ level. Plant organs often have small and intricate structures; for example, a 1.5meter-tall sapling may have branches with diameters as small as 2 millimeters.Existing NeRF algorithms have limitations in representing fine geometric details. To address this issue, we propose three steps: a novel ray sampling strategy, Hash encoding to map input points to a higher-dimensional space, and an optimization framework to refine camera poses.
3. Removing image backgrounds enhances plant reconstruction but obtaining masks in natural scenes is challenging. Traditional solid-colored studio setups are impractical for “plant-to-camera” modes. We propose a ray sampling strategy integrated with prior knowledge in OB-NeRF to improve reconstruction without plant masks.
4. A significant hurdle in reconstruction under natural scene is the presence of uneven spatial lighting. To address this issue, we incorporate an exposure adjustment phase into OB-NeRF.

**Figure 5:**
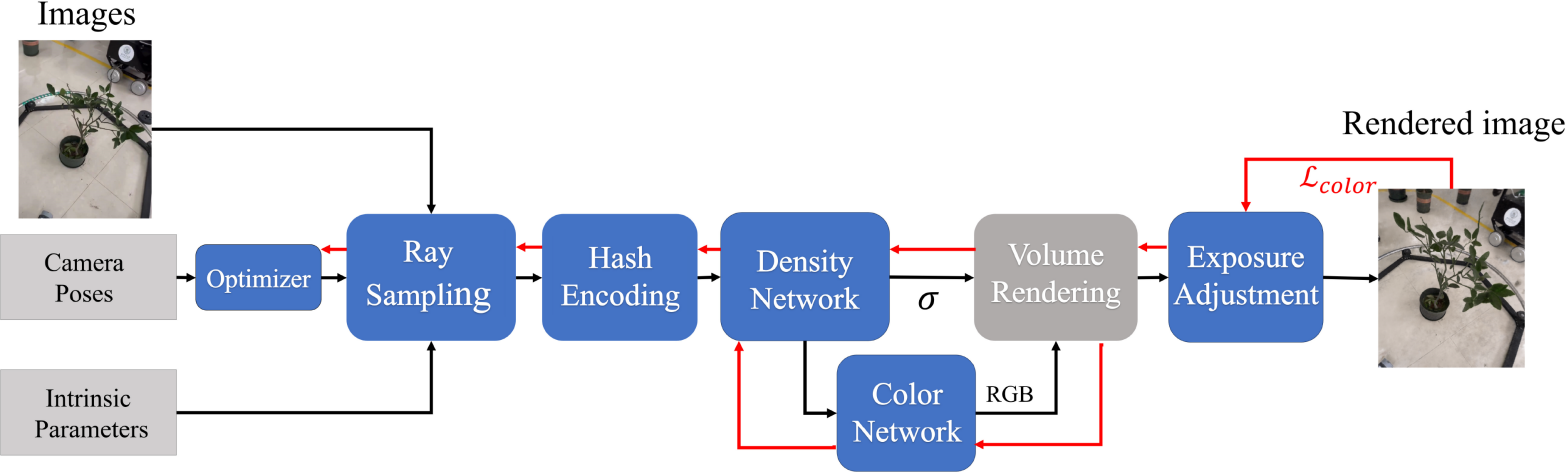
The pipeline of OB-Nerf.

These strategies result in the creation of the OB-NeRF model, as shown by its internal structure in **Figure 5**.

##### 3.3.3.1 Ray sampling strategy based on region of Interest

To optimize the neural radiance field, the first step is to generate a set of sampled 3D points along the camera rays passing through each pixel of the input images. The scene can be divided into ROI and background. ROI is the area where the target plant for reconstruction is located and is dense in volume in space. The quality of the neural field in ROI determines the reconstruction’s quality in the target plant. Moreover, there are many empty spaces in the scene. Sampling points falling in these empty spaces do not contribute to the optimization process. Instead, they increase computational and memory overhead. We utilize a hierarchical sampling approach, and optimize two networks to reconstruct the scene: a “coarse” network and a “fine” network, with the output of the “coarse” network guiding the sampling point selection for the “fine” network. Therefore, we implement a new ray sampling strategy that focuses the neural field optimization process on the ROI area and skips the empty spaces, thereby improving the reconstruction quality of the target plant and reducing reconstruction time.

First, we calculate the intersection point between the ray with origin *o* = (*o_x_, o_y_, o_z_*) and direction *d* = (*d_x_, d_y_, d_z_*) and the ROI’s AABB. The formula is presented in Equation 8:


(8) $$\left\{ \begin{array}{l} t_{\text{min}}=\text{max }(\frac{x_{\text{min}}-o_{x}}{d_{x}},\frac{y_{\text{min}}-o_{y}}{d_{y}},\frac{z_{\text{min}}-o_{z}}{d_{z}})\\ t_{\text{max}}=\text{min }(\frac{x_{\text{max}}-o_{x}}{d_{x}},\frac{y_{\text{max}}-o_{y}}{d_{y}},\frac{z_{\text{max}}-o_{z}}{d_{z}}) \end{array}\right.$$

Where: *t_max_* and *t_min_* represent the distance from the intersection point to the origin of the ray. The ray intersects the AABB if and only if *t*_min_ ≤ *t*_max_ and *t*_max_ ≥ 0. Because the camera imaging position is contained within the AABB, *t*_min_ ≤ 0. We assign a value of 0 to *t*_min_.

**Figure 6:**
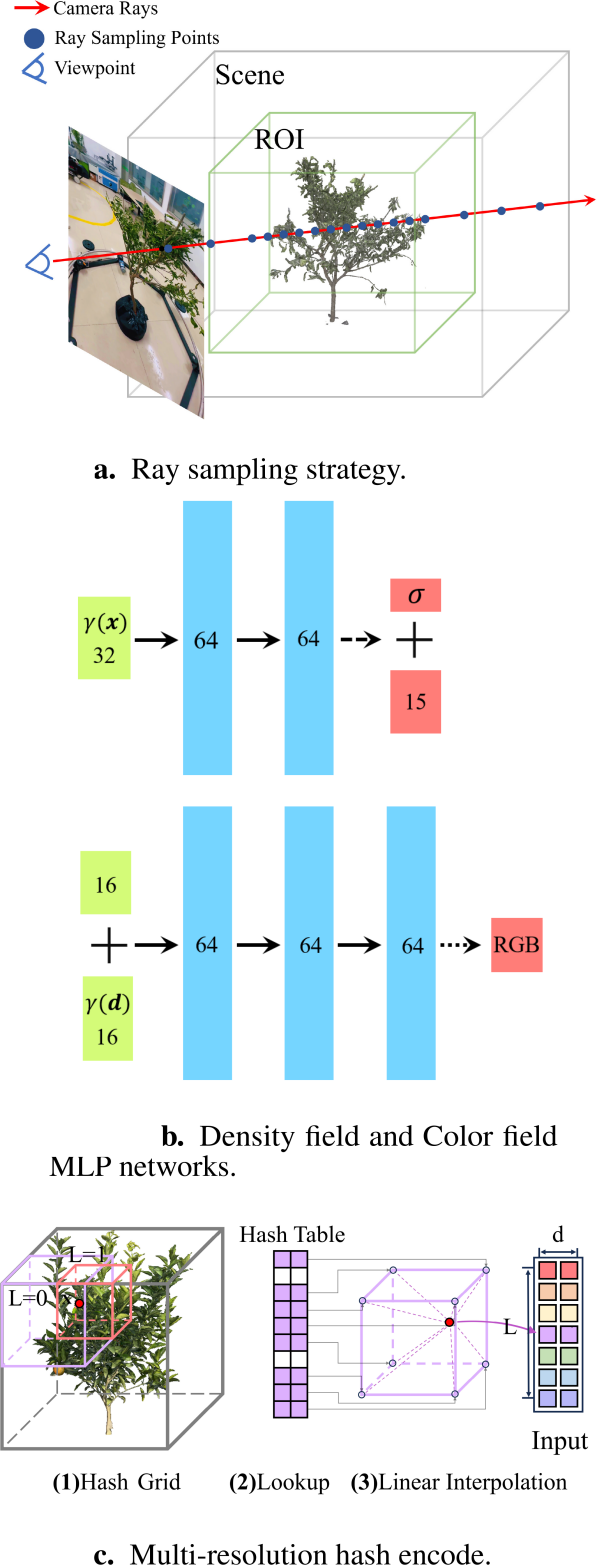
Improvement strategies of OB-NeRF. **(a)** Ray sampling strategy. **(b)** Density field and Color field MLP networks. **(c)** Multi-resolution hash encode.


Secondly, the intersection point divides the ray into two regions, (0*, t_max_*) and (*t_max_, t_b_*), where *t_b_* represents the maximum length of the ray. We generate sampling points with different sampling intervals in two regions and obtain a total of *N_c_* sampling points(**Figure 6a**). Thirdly, we obtain the output *ω_i_* by inputting the *n_c_* sampling points into the coarse network, as illustrated in Equation 9, and normalization is applied to *ω_i_* to determine the respective constant probability density function ${\hat{\omega }}_{i}=\frac{\omega_{i}}{\sum_{j=1}^{N_{c}}\omega_{j}}$ in two regions. Subsequently, total *N_f_* points are obtained through inverse transform sampling.

(9) $$\omega_{i}=T_{i}(1-\text{exp}(\sigma_{i}\delta_{i})),\text{where}T_{i}=\text{exp }(-\sum_{j=1}^{i-1}\sigma_{j}\delta_{j})$$

where $\delta_{i}=t_{i+1}-t_{i}$ is the distance between adjacent samples

Finally, we combine *N_c_* and *N_f_* points as “fine” network input points.

##### 3.3.3.2 Shallow MLP

To enhance the speed of the 3D reconstruction pipeline, we employ shallow MLP networks for approximating the density and color fields. The architecture of the two fully connected networks is illustrated in **Figure 6b**, input vectors are represented by green rectangular blocks, intermediate hidden layers by blue rectangular blocks, and output vectors by red rectangular blocks. The number within each block indicates the vector’s dimension. All layers consist of standard fully connected layers. Black arrows signify layers with ReLU activation, dashed black arrows represent layers with sigmoid activation, and dotted black arrows with a black dot indicate layers with ELU activation. The “+” symbol denotes vector concatenation. The density network takes the positional encoding of the input location and produces a volume density and a 15-dimensional geometric feature vector. The color network takes the 16-dimensional geometric feature along with the positional encoding of the viewing direction to output RGB values.

##### 3.3.3.3 Multi-resolution hash encode

We employ a shallow multi-layer perceptron to fit color and density fields. However, shallow MLP tends to learn low-frequency functions, resulting in poor reconstruction of high-frequency details in color and geometric shapes, failing to effectively represent the plant organs. To address this issue, the study adopted multi-resolution hash techniques from Instant-NGP (Müller et al., 2022) for position encoding (**Figure 6c**). The hash table parameters are shown in **Table 1**. In terms of implementation details, to avoid increasing the computational load by simply augmenting the number of hash table layers, this study optimizes the design of the hash table. In the final level of the hash table, space is divided into 2048 hash voxels instead of the conventional 512. This design does not alter the dimension of the hash encoding output. Moreover, it enhances the encoding capacity at finer scales, thereby improving the fine-scale reconstruction performance of OB-NeRF.

**Table 1:** Hash table parameters.

Parameter	Symbol	Value
Number of levels	*L*	16
Max. entries per level (hash table size)	*T*	219
Number of feature dimensions per entry	*F*	2
Coarsest resolution	*N*_min_	16
Finest resolution	*N*_max_	2048

##### 3.3.3.4 Exposure adjustment

In natural environments, scene lighting conditions are not uniform, leading to varying exposure levels in images from different perspectives. Conventional 3D reconstruction often involves setting up controlled lighting environments to provide stable and uniform lighting. To achieve 3D reconstruction under uncontrolled lighting conditions, this study incorporates the exposure rate *E_i_* of each image as a learnable parameter into the optimization process, incorporating the exposure adjustment into the forward propagation. By adjusting color values during image synthesis with the learned exposure rates, the study compensates for the exposure variations in images. Through the backpropagation algorithm, the study dynamically updates the exposure rate for each image based on gradient information from the loss function. An L2 regularization term is utilized to supervise the optimization of exposure rates to prevent overfitting. With the exposure adjustment step, we substantially enhance the accuracy of 3D reconstruction under uneven lighting conditions and improve the algorithm’s robustness to complex and variable lighting scenarios.

In our proposed method, the pixel values of the synthesized images are adjusted by calculating the scaling factors *S_i_* corresponding to the exposure rate *E_i_* of each image. The exposure rate *E_i_*, *i* represents the exposure level of image *i*, while the scaling factor *S_i_* is a multiplicative factor used to adjust the pixel values of the synthesized image *i*. The adjustment of pixel values can be expressed as Equations 10 and 11:


(10) $$S_{i}=e^{\text{ln}(2)\times E_{i}}$$

(11) $$RGB_{\text{target}}=S_{i}\cdot RGB$$

##### 3.3.3.5 Camera pose optimizer

Precise camera pose ensures the accurate transformation from the world coordinate system to the camera coordinate system, serving as the basis of the entire rendering and training process. Although we have already globally calibrated the camera poses, we have observed potential minor deviations between the calibrated camera poses and their true values on the level of a single image. In this study, we construct a camera pose optimizer for each image that incorporates two trainable parameters: the camera’s position offset and rotation offset. The optimizer, integrated into the network’s forward propagation process, iteratively updates these parameters based on the loss function using the backpropagation algorithm. An L2 regularization term is utilized to supervise the optimization of the camera pose to prevent overfitting.

#### 3.3.4 Mesh extraction from neural radiance fields

NeRF is an implicit continuous representation of the 3D scene. To discretely sample the radiance field and generate a mesh model, we employ the Marching Cubes algorithm, a classic method to extract iso-surfaces from volumetric data by approximating the surface with a polygonal mesh.

Firstly, Given the predefined 3D ROI, a set of spatial points *P* = {*p*_1_ *, p*_2_ *,…, p_n_*} is generated via dense volumetric sampling. To represent the region of interest more accurately, we use the inscribed cylinder of the AABB as the sampling area. Secondly, For each point *p_i_* ∈ *P*, using the OB-NeRF model to obtain the density values, *σ*(*p_i_*) = NeRF*σ*(*p_i_*). Finally, The Marching Cubes algorithm (Lorensen and Cline, 1987) identifies the isosurface by thresholding the density values, as illustrated in Equation 12:


(12) $$M=\text{MarchingCubes}(P,\sigma_{\text{threshold}})$$

Where: *M* is the resultant mesh and *σ*_threshold_ is an optimal density value demarcating the object’s boundary.

## 4 Result and discussion

### 4.1 Evaluation of 3D reconstruction performance

In this section, we employed our 3D reconstruction platform to reconstruct twenty saplings from three size categories, representing distinct growth stages, for verifying platform performance. This categorization ensures a comprehensive analysis across the growth stages. After acquiring the sapling mesh models, We extracted the plant height from the Mesh model and used CloudCompare software to measure leaf length and width. In addition, we manually measured plant height, leaf length, and leaf width.

We evaluate the 3D reconstruction performance of our platform using the PSNR of the generated synthetic images, along with the accuracy, spatial resolution, and texture resolution of the generated mesh models.

**Figure 7:**
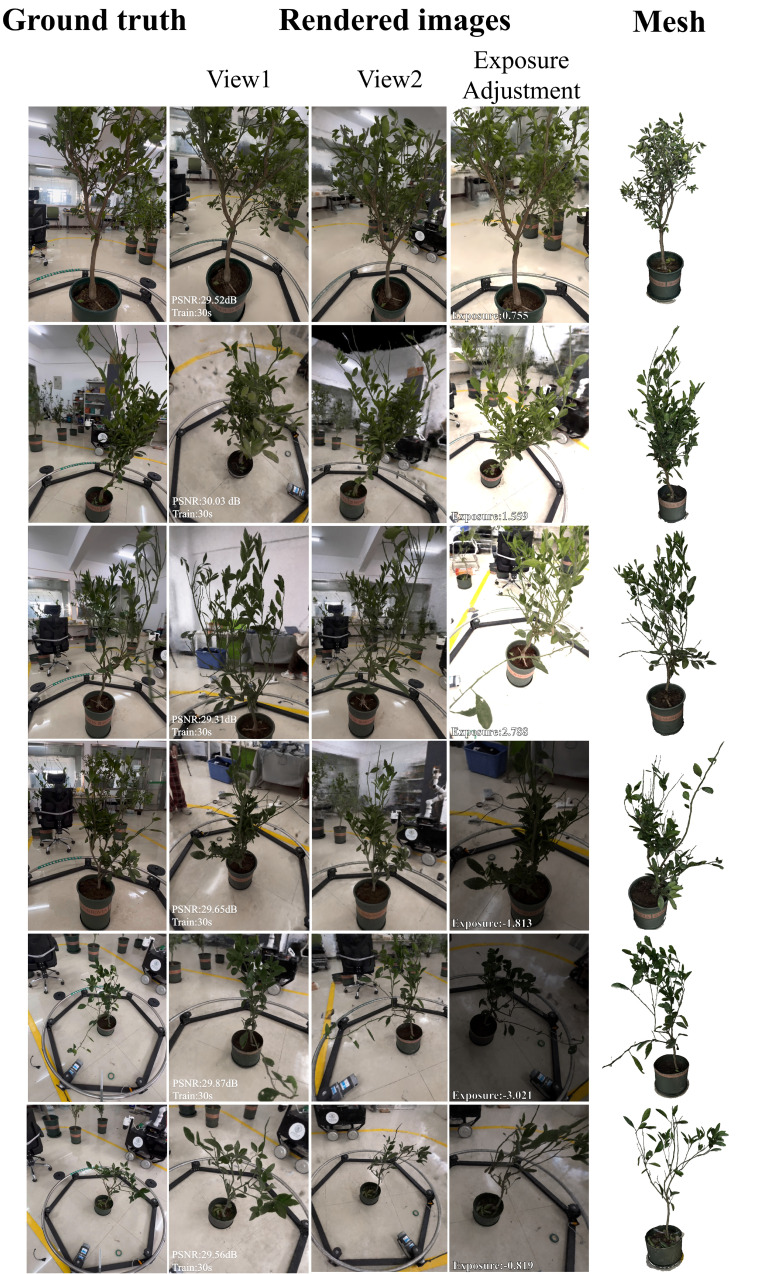
3D reconstruction results.


Peak Signal-to-Noise Ratio (PSNR) is a widely recognized quantitative metric for assessing image quality. Higher PSNR values denote better image fidelity and closer similarity to the original image. We utilized PSNR to evaluate the quality of the synthesized novel views, with PSNR calculations based on the Mean Squared Error (MSE), which quantifies the average squared difference in pixel values between the reference image and the image under assessment. Its calculation method is shown in Equations 13 and 14. As depicted in **Figure 7**, we present the rapid reconstruction capability and exceptional effects of the OB-NeRF algorithm, as evidenced by the synthesized images and extracted 3D mesh models. We employ PSNR as the metric to evaluate the quality of synthesized images of target plants. **Figure 7** also demonstrates our capability in exposure adjustment, with the exposure values indicating deviations from the original exposure rate. These results were achieved after merely 30 s of OB-NeRF training, substantiating the proposed algorithm’s significant speed advantage. By introducing a new ray sampling strategy, we embedded prior knowledge into OB-NeRF, enabling the algorithm to reconstruct target plants amidst complex backgrounds efficiently. Notably, the analysis indicates that the target plants can still be reconstructed with high fidelity (PSNR = 29.95 dB), even when the background reconstruction exhibits significant distortion (PSNR *<* 20 dB).

(13) $$\text{MSE}=\frac{1}{MN}\sum_{i=1}^{M}\sum_{j=1}^{N}{\left[I_{\text{real}}(i,j)-I_{\text{synthesized}}(i,j)\right]}^{2}$$

(14) $$\text{PSNR}=10\cdot \log_{10}(\frac{{\text{MAX}}_{i}^{2}}{\text{MSE}})$$

**Figure 8:**
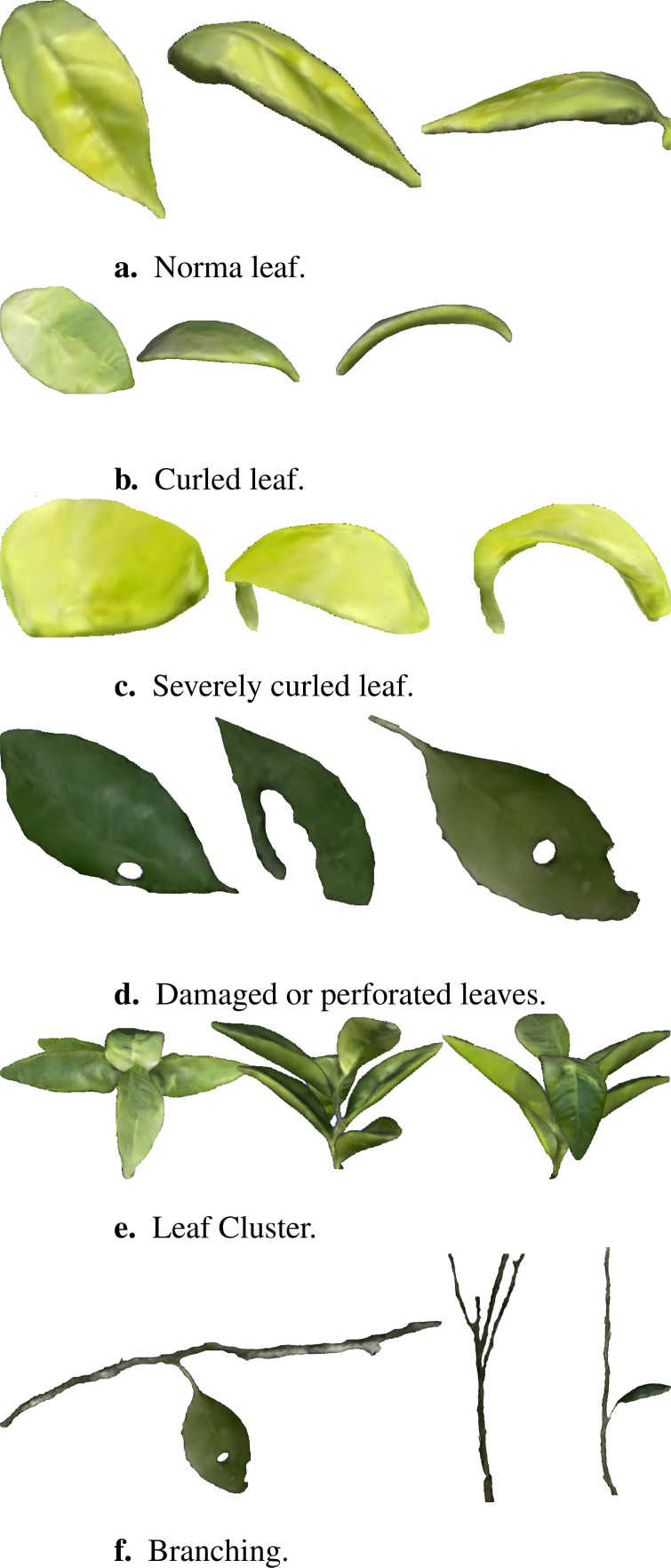
Organ-level reconstruction results. **(a)** Norma leaf. **(b)** Curled leaf. **(c)** Severely curled leaf. **(d)** Damaged or perforated leaves. **(e)** Leaf Cluster. **(f)** Branching.

As demonstrated in **Figure 8**, we present different organs of the plant mesh model, including normal leaves, curled leaves, damaged or perforated leaves, leaf clusters, and branches. The average reconstruction error of these organs is less than 2mm, and the texture resolution reaches 0.5 mm/pixel, exhibiting high geometric and texture fidelity. The reconstruction results demonstrate that our platform also has good reconstruction capabilities at the organ level of plants and validates its applicability and efficiency in handling plants with varying growth states and complexity.

**Figure 9:**
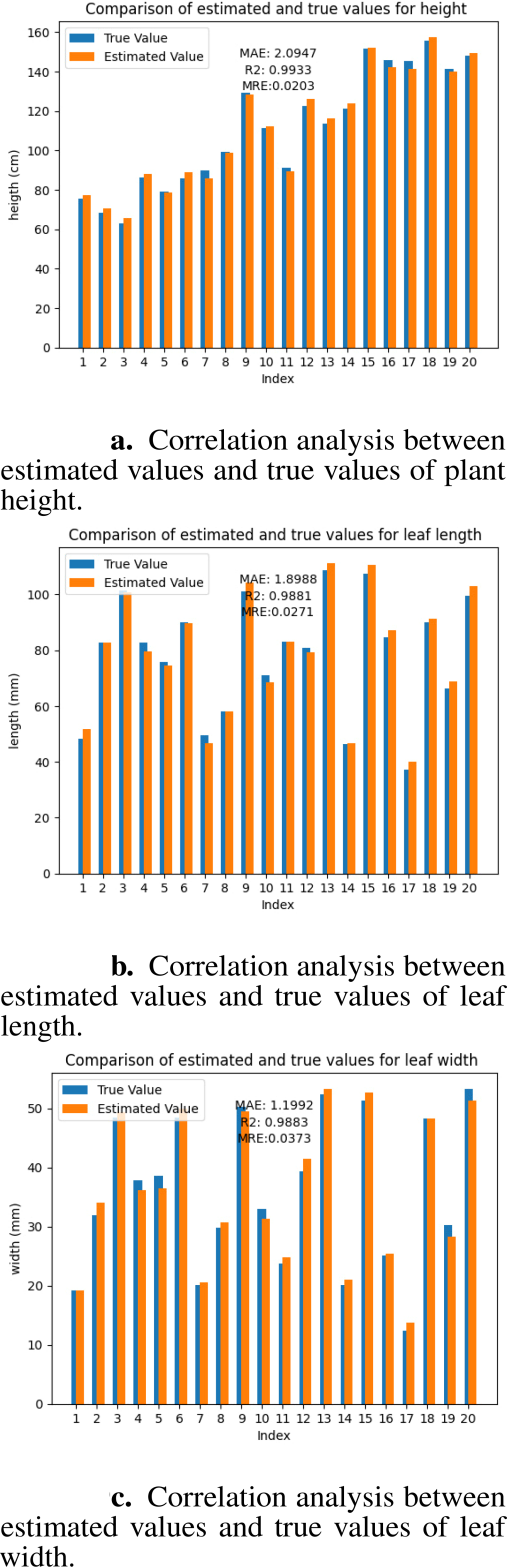
Correlation analysis between estimated values and true values. **(a)** plant height. **(b)** leaf length. **(c)** leaf width.

The accuracy of the mesh model directly reflects the quality of the geometric reconstruction. To verify the precision of the 3D reconstruction platform developed in this study, we selected three key phenotypic parameters: tree height, leaf length, and leaf width, and analyzed the error between their estimated and true values. These parameters can reflect the overall growth status and leaf development level of seedlings and are important indicators for evaluating seedlings’ phenotypes. The estimated value of the tree height is obtained by calculating the difference between the maximum and minimum values of the model on the z-axis. We manually measured each seedling using a tape measure to obtain the true values. Each fruit tree was measured three times, and the average value was taken as the final true value. The estimated leaf length and width values were obtained using the CloudCompare software. We converted the Mesh model into a point cloud and manually segmented the target leaves. In the measurement tool of CloudCompare, we selected the points at both ends of the long axis and short axis of the leaf, and the software automatically calculated the Euclidean distance between the two points, which were used as the estimated values of leaf length and leaf width. For the measurement of true values, we picked the target leaves and measured them three times each in the long-axis and short-axis directions using a vernier caliper (accuracy 0.01 mm), and the average values were taken as the true values of leaf length and leaf width. We used mean absolute error and coefficient of determination to evaluate the accuracy of the reconstruction results. MAE reflects the average size of the deviation between the estimated and true values, with smaller values indicating smaller deviations and 0 being the ideal value. *R*_2_ reflects the goodness of fit between the estimated and true values, ranging from 0 to 1, with values closer to 1 indicating a higher goodness of fit. The MAE of tree height was 2.0947 cm, with an *R*_2_ of 0.9933 (**Figure 9a**); the MAE of leaf length was 0.1899 cm, with an *R* ^2^ of 0.9881 (**Figure 9b**); and the MAE of leaf width was 0.1199 cm, with an *R* ^2^ of 0.9883 (**Figure 9c**). Experimental results demonstrate that our method achieves reconstruction accuracy at the millimeter level. The results demonstrate that our reconstruction platform has high accuracy at both the individual plant and organ levels.

Spatial resolution represents whether the model retains sufficient detail information, while texture resolution affects the perceived visual quality of the model. The spatial resolution reached 0.0019 mm, and the texture resolution reaches 0.5 mm/pixel, exhibiting high geometric and texture fidelity.

### 4.2 Ablation experiment

#### 4.2.1 Correlation between the numbers of images and SFM reconstruction time consumption

**Figure 10:**
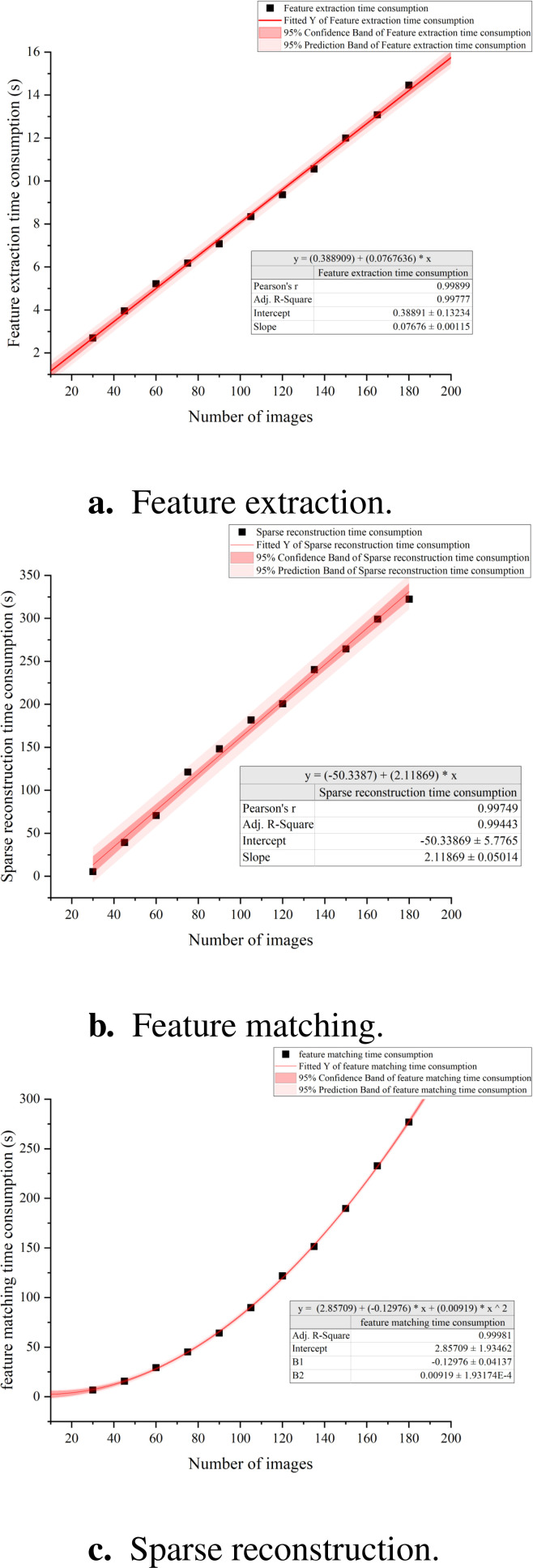
Correlation between SFM time consumption and the number of images. **(a)** Feature extraction. **(b)** Feature matching. **(c)** Sparse reconstruction.

To reduce the time consumption of the 3D reconstruction pipeline, we investigated the impact of the number of images on the time consumption using the SfM method. We established experimental groups with varying numbers of images, specifically 45, 60, 75, 90, 105, 120, 135, 150, 165, and 180 images. Each group included six fruit trees of different sizes as experimental subjects. To minimize random errors in the results, each fruit tree underwent 10 independent SfM reconstructions, resulting in a total of 60 reconstructions per group (10 reconstructions × 6 trees). We recorded the time taken for each reconstruction and calculated the average reconstruction time for each group to assess the relationship between the number of images and reconstruction duration. Ultimately, we employed Origin software for data analysis to quantitatively explore the specific impact of the number of images on reconstruction time. This analysis aided us in understanding how to select the optimal number of images to optimize the efficiency of the entire reconstruction process while ensuring the quality of the reconstruction.

we conducted a systematic analysis of the time required for the three primary steps in Structure from Motion method: feature extraction, feature matching, and sparse reconstruction. Our findings reveal that for datasets ranging from 30 to 180 images, the time needed for feature extraction scales linearly with the number of images and represents a smaller fraction of the overall processing time (**Figure 10a**). In contrast, the time dedicated to feature matching increases quadratically as the number of images grows, accounting for the largest portion of the total processing time (**Figure 10b**). Regarding the sparse reconstruction phase, while its time consumption also follows a linear trend with the image count, this stage exhibits considerable variability in individual reconstruction efforts (**Figure 10c**).

To establish a high-throughput 3D reconstruction pipeline, it is crucial to minimize the number of images used while ensuring the reconstruction’s quality is not excessively compromised.

#### 4.2.2 Correlation between the numbers of images and reconstruction quality

**Figure 11:**
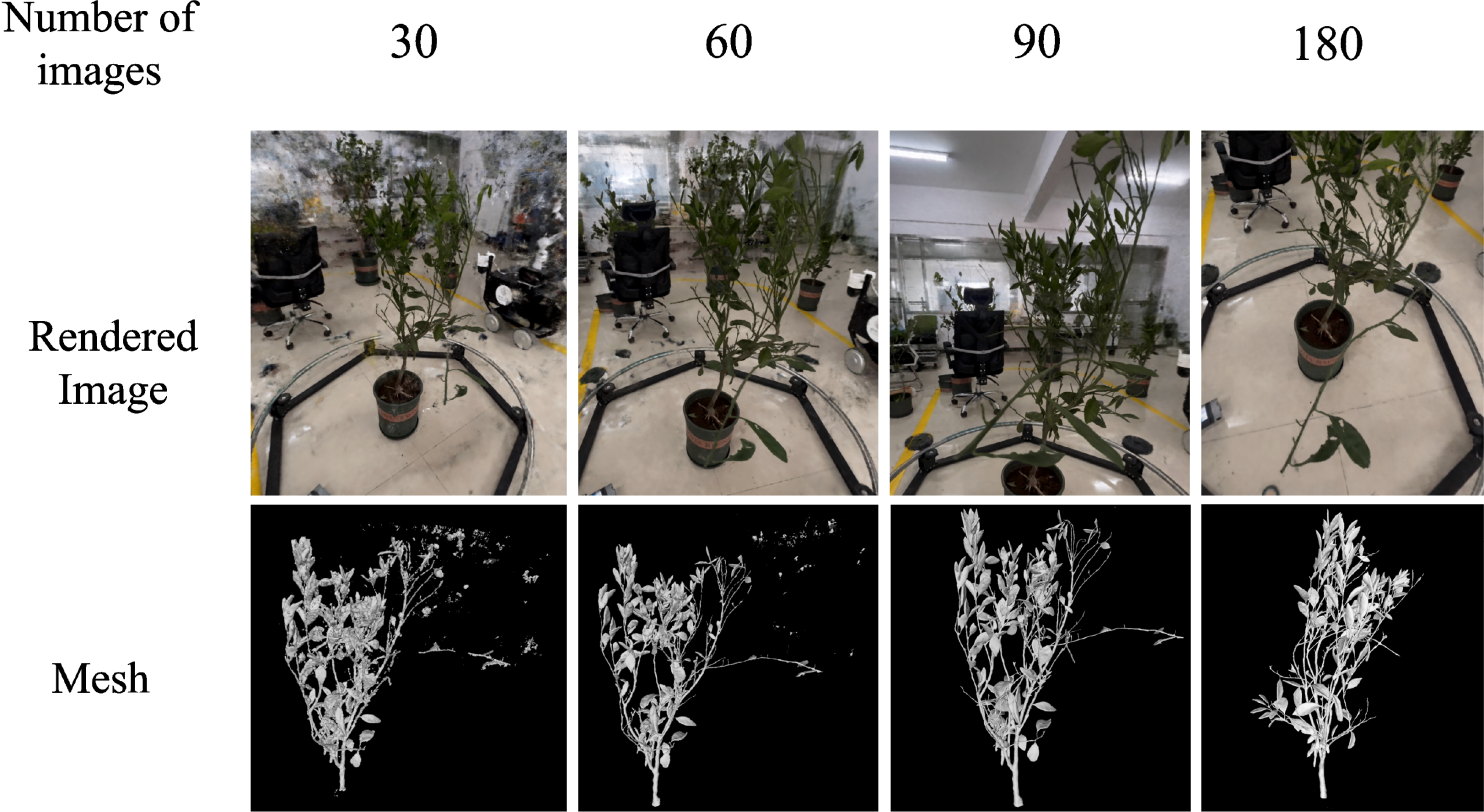
Correlation between the numbers of images and reconstruction quality.

We assess the quality of the reconstruction from two aspects: the synthesized images and the extracted mesh models. To more intuitively represent the geometric shape of the mesh, we opt to display it using an uncolored mesh. This approach enhances the clarity of its structural details. **Figure 11** clearly illustrates the relationship between the number of images and the quality of reconstruction. The analysis indicates that a relatively high quality of reconstruction is already achieved with 90 images. However, when the number of images is increased to 180, there is no significant improvement in reconstruction quality. We conclude that selecting 90 images as the input for the reconstruction pipeline is optimal. When processing 90 images, SFM takes about 210s, the pipeline takes about 250s.

#### 4.2.3 Assessment of training efficiency and reconstruction quality for OB-NeRF

To accelerate the training speed and enhance the reconstruction quality of the NeRF model for the target plant, we employed an optimized multi-resolution hash encoding, a shallow MLP network, exposure adjustments, camera extrinsics optimization, and a new ray sampling strategy. We designed comparative experiments between OB-NeRF, Based-NeRF, Mip-NeRF (Barron et al., 2021), Neus (Wang et al., 2023a), NeRFacto (Tancik et al., 2023) and Instant-NGP. For Neus, we use an accelerated version based on multiresolution hash encoding and open-source library NerfAcc (Wang et al., 2023b). The results, as shown in **Table 2**, indicate that the training time requirement for Based-NeRF ranged between 9 to 12 hours, whereas OB-NeRF significantly reduced training time. Compared with Mip-NeRF,NeRFacto and Neus, our method shows advantages in both efficiency and reconstruction quality. Compared to Instant-NGP, which also utilizes multi-resolution hash encoding, our network demonstrated a faster training speed and reconstruction quality in reconstructing target plants.

**Table 2:** Comparison between Based-NeRF, Mip-NeRF, Instant-NGP, Neus, NeRFacto and Ours.

Method	Average Training Time	Average PSNR
Based-NeRF	10 h	24.71dB
Mip-NeRF	3 h	25.81 dB
Instant-NGP	30 s	26.79 dB
Neus	30min	27.31dB
NeRFacto	5min	27.64dB
Ours	30 s	29.95 dB

We compared the geometric reconstruction performance of NeRFacto, Instant-NGP, NeuS, and our method, with the results shown in **Tables 3** and **4**. Experimental results demonstrate that our method achieves higher reconstruction accuracy than NeRFacto and Instant-NGP, and is comparable to NeuS. However, NeuS requires significantly more reconstruction time, indicating lower efficiency.

**Table 3:** H measurement results of NeRFacto, Instant-NGP, Neus and Ours.

Method	*R* ^2^	MRE (%)	MAE (cm)
Instant-NGP	0.963	2.60	2.67
Neus	0.992	2.05	2.11
NeRFacto	0.987	2.47	2.54
Ours	0.993	2.03	2.09

**Table 4:** Leaf width measurement results of NeRFacto, Instant-NGP, Neus and Ours.

Method	*R* ^2^	MRE (%)	MAE (cm)
Instant-NGP	0.947	4.20	0.2357
Neus	0.990	2.15	0.1208
NeRFacto	0.961	2.84	0.1593
Ours	0.988	2.14	0.1199

### 4.3 Comparison with other 3D reconstruction methods

Conventional image-based plant 3D reconstruction require the acquisition of plant masks to accelerate reconstruction speed and enhance the quality of the reconstruction. Moreover, they are sensitive to lighting conditions, necessitating stable and uniform illumination. However, achieving these conditions in real scenarios is challenging. Our 3D reconstruction platforms accomplish 3D reconstruction of small to medium-sized plants against complex backgrounds without requiring plant masks. Additionally, it is robust to variations in lighting, eliminating the need for stable, uniform illumination.

**Figure 12:**
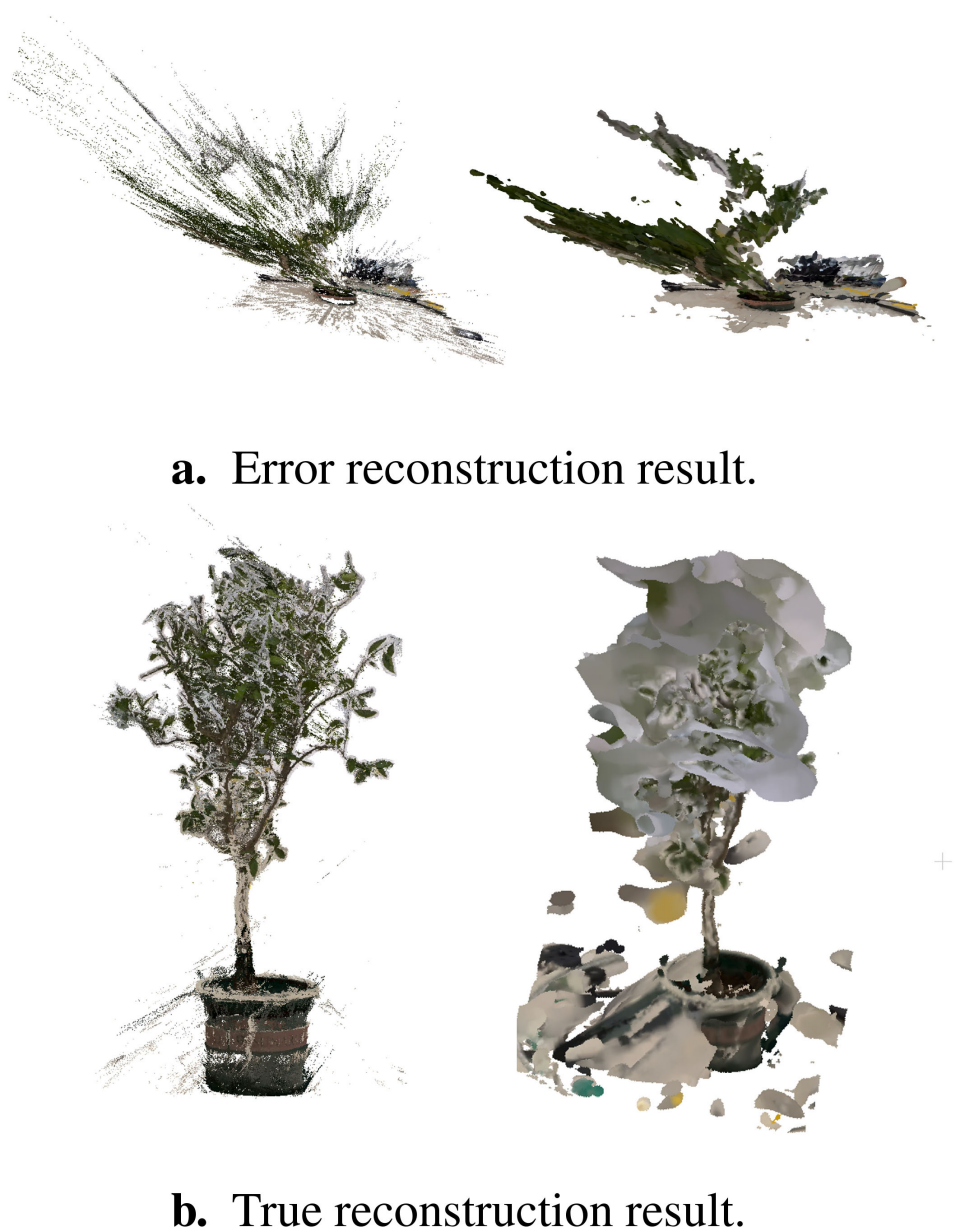
The reconstruction result of COLMAP. **(a)** Error reconstruction result. **(b)** True reconstruction result.

The mainstream algorithm for image-based 3D reconstruction is SfM-MVS. COLMAP is widely recognized as an advanced and efficient tool for implementing SfM-MVS. We utilized COLMAP to reconstruct the same datasets. As illustrated in **Figure 12a**, certain saplings underwent incorrect reconstruction. In contrast, **Figure 12b** demonstrates that even the correctly reconstructed saplings exhibit low-quality results. The point cloud derived from the reconstruction process contains numerous floating noise points, and severe artifacts are evident along the edges of the branches and leaves. These issues contribute to the generation of low quality meshes. Furthermore, the reconstruction time using COLMAP exceeds two hours, whereas our algorithm completes the reconstruction in less than five minutes.

**Figure 13:**
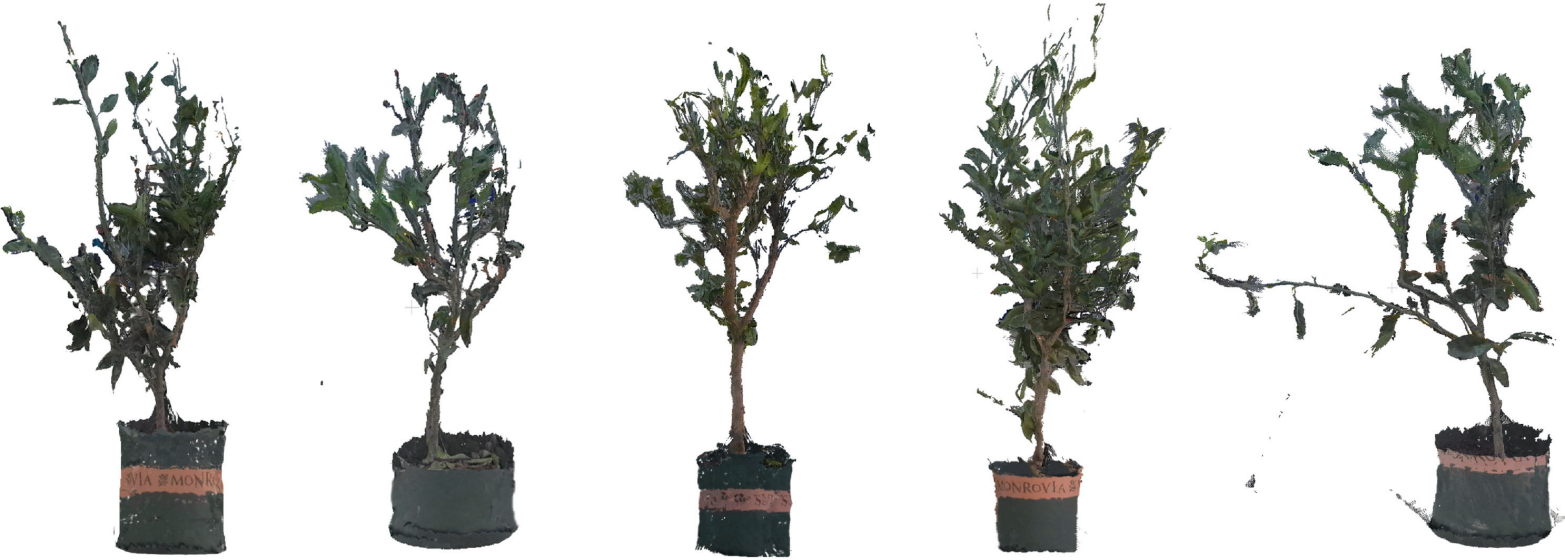
The reconstruction result of Kinect.

In recent years, the field of 3D reconstruction of plants has seen the emergence of Kinect as a novel trend. Our laboratory previously experimented with the use of Kinect for quad-view imaging, combined with Iterative Closest Point (ICP) registration techniques, successfully reconstructing a 3D model of a single rapeseed (Teng et al., 2021b). We subsequently attempted to apply this technique to the 3D reconstruction of saplings(**Figure 13**). However, for saplings with complex structures and larger sizes, the reconstruction results obtained with Kinect were not satisfactory. We observed that the point clouds reconstructed using Kinect exhibit significant data gaps in many areas, particularly in sections with complex structures. The point clouds are heavily affected by noise, and artifacts appear at the edges of branches and leaves. These issues prevent the reconstructed models from accurately reflecting the precise geometric structure of the plants. During the ICP registration process, we also encountered several instances of failure.

**Figure 14:**
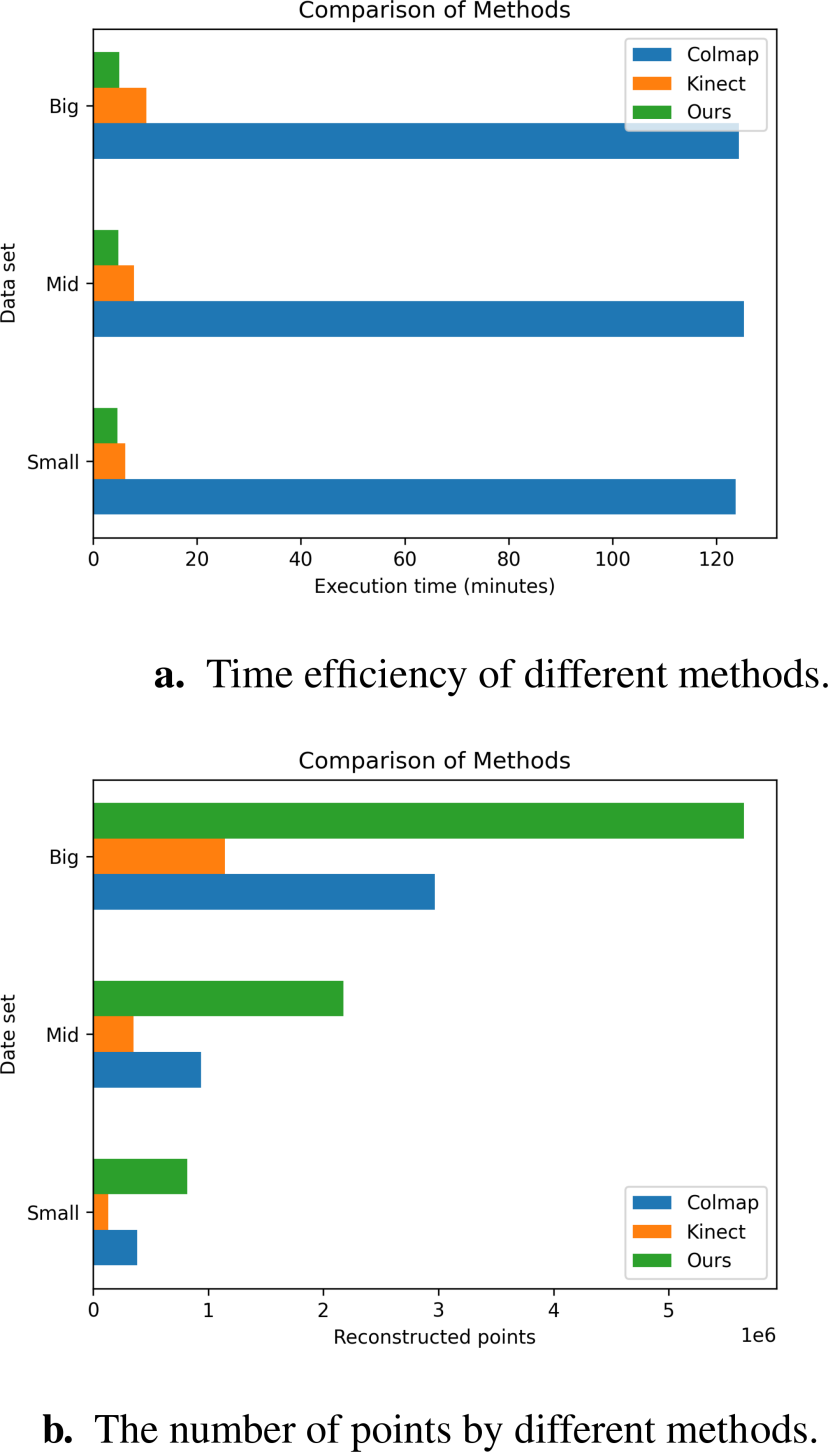
Comparison of objective metrics for different methods on different data sets. **(a)** Time efficiency of different methods. **(b)** The number of points by different methods.

**Figure 15:**
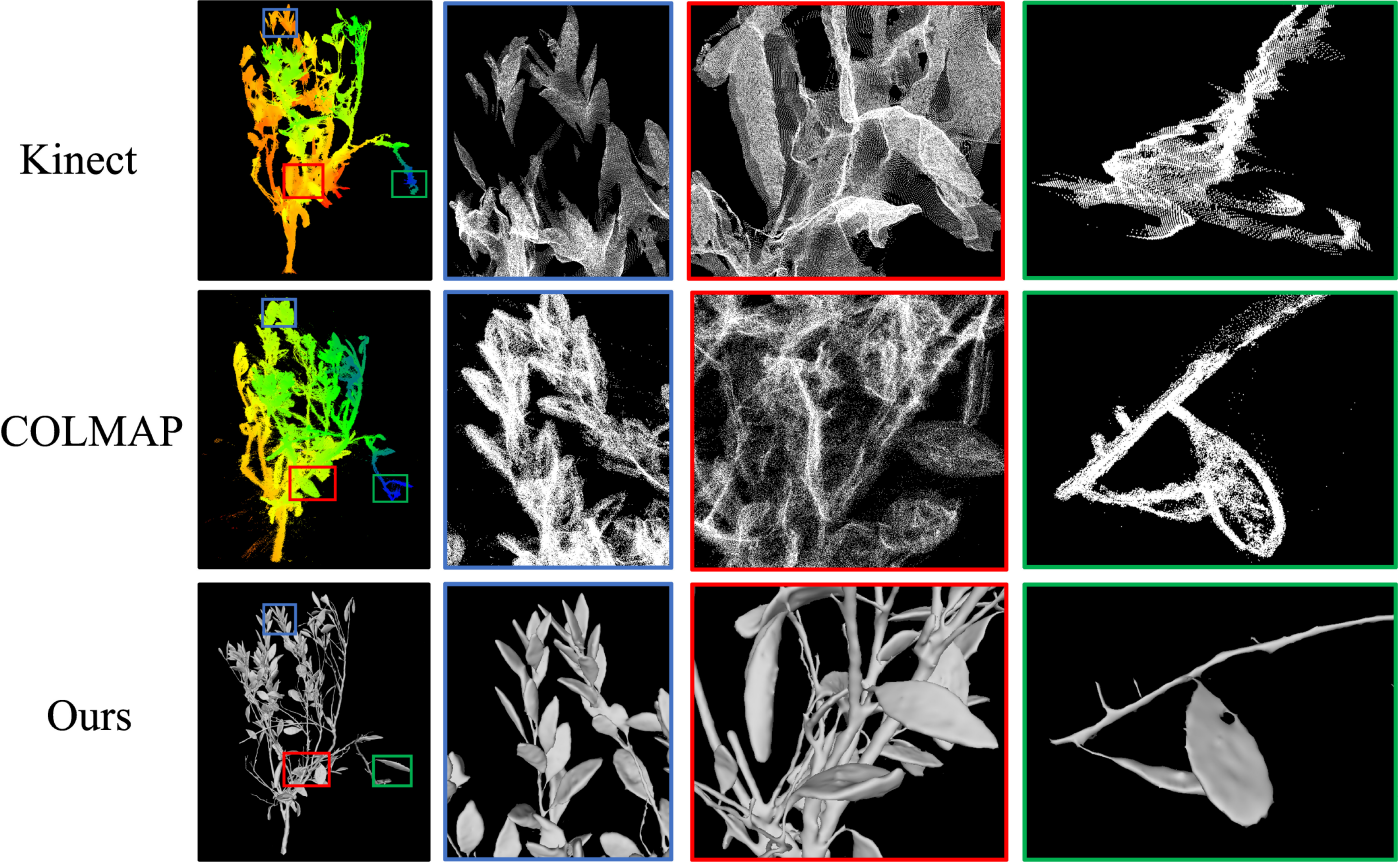
Comparison of the reconstruction results for Kinect, COLMAP, and Ours.

Objective metrics evaluate the performance of reconstruction algorithms by considering the number of points, the Spatial resolution, and time efficiency. **Figure 14** and **Table 5** show the result of the three methods’ average performance on the same metrics. In **Figure 15**, we intuitively show the comparison of the reconstruction results of the three methods. The reduction in time consumption achieved by our method, presented in **Figure 14a**, is substantial. Specifically, our method reduces time consumption by 96.08% relative to Colmap, and 49.73% relative to Kinect-based These findings underscore the high time efficiency of the algorithm proposed in this paper. As delineated in **Figure 14b**, The model’s points generated by our method outperform Colmap by 159.26% and Kinect-based by 393.72%. As shown in **Table 5**, the spatial resolution of the model generated by our method is 0.19 mm, which is much smaller than that of Colmap and Kinect-based methods and has stronger geometric detail representation capabilities. As shown in **Table 6**, we also present the comparison of our tree seedling height measurement results with other literature.

**Table 5:** Spatial resolution of three methods.

Method	Spatial resolution
Colmap	0.0100mm
Kinect	0.0037mm
Ours	0.0019mm

**Table 6:** H measurement results of our platform and other literature.

Plant	*R* ^2^	MRE(%)	MAE(cm)
Tree seedlings(Ours)	0.993	2.03	2.09
Seedlings (Yang et al., 2022)	0.991	2.79	2.72
lower plants (Wu et al., 2022)	0.991	–	–
Fruit tree (Yang et al., 2019)	0.960	2.50	–
Tree (Li and Tang, 2017)	0.982	–	–

## 5 Conclusions

A 3D reconstruction platform based on multi-view images is proposed to reconstruct complex plants, comprising three steps: multi-view images acquisition, estimation, and calibration of camera parameters, and 3D reconstruction using OB-Nerf. In our study, to address the issue of model deformation during the reconstruction process, camera poses are automatically calibrated using the imaging trajectories as priori information, eliminating the need for additional calibrators. Furthermore, we propose OB-NeRF, an innovative Nerf-based algorithm for 3D reconstruction. The algorithm incorporates an optimizer of camera poses and an exposure adjustment mechanism. a new ray sampling strategy is introduced. It employs multi-resolution hash encoding techniques in conjunction with a shallow MLP network. Furthermore, the algorithm features an automated strategy for Mesh model extraction. OB-NeRF can reconstruct high-quality neural radiation fields of complex plants from images acquired under complex backgrounds and uneven illumination within 30 seconds. Subsequently, it accomplishes the synthesis of images and the automatic extraction of Mesh. From the perspectives of novel viewpoint image synthesis and mesh modeling, our results have exhibited outstanding texture quality and geometric fidelity at the levels of both individual plants and their respective organs. The average PSNR of the synthesized images is 29.97dB, and the spatial resolution of the Mesh model is 0.19 mm. By comparing the three phenotypic parameters estimated from the model—tree height, leaf length, and leaf width—with manually measured values, the Mean Square Errors obtained were 2.0947 cm,0.1898 cm, and 0.1199 cm, respectively. The coefficients of determination were 0.9933,0.9881 and 0.9883, leading to a robust linear relationship between the extracted phenotype and measured traits.

In summary, the proposed low-cost reconstruction platform is capable of completing data acquisition for an individual plant in approximately 15 seconds and can perform high-quality reconstruction of the collected data within 250 seconds. The developed platform lays a solid foundation for the application of high-throughput phenotyping and digital twins in agriculture. It shows great potential in accelerating plant breeding, enabling precise crop management, and facilitating plant growth monitoring.

In the future, we plan to extend our work in the following directions:

1. Deploying the 3D reconstruction process to the cloud and achieving communication with the image acquisition system. This will improve the scalability, accessibility, and processing speed of our approach, making it more suitable for large-scale applications.
2. Leveraging the concept of collaborative design of software and hardware to optimize the 3D reconstruction pipeline further. By employing precise mechanical design and motor control, we aim to rotate and translate the camera under known poses accurately. This hardware-based approach will enable us to quickly obtain camera poses, replacing the need for the SFM method.
3. Our method is primarily designed for the 3D reconstruction of individual plants. However, when extended to large-scale field scenarios, experimental results revealed its performance limitations. To address this issue, future work could consider introducing new optimization strategies or adopting more advanced 3D reconstruction algorithms, such as 3DGS.

## Data Availability

The raw data supporting the conclusions of this article will be made available by the authors, without undue reservation.
